# Sensitization profile to sawtooth oak component allergens and their clinical implications

**DOI:** 10.1002/jcla.23825

**Published:** 2021-05-18

**Authors:** Kyoung Yong Jeong, Jongsun Lee, Min Kyu Sang, Yong Seok Lee, Kyung Hee Park, Jae‐Hyun Lee, Jung‐Won Park

**Affiliations:** ^1^ Department of Internal Medicine Institute of Allergy Yonsei University College of Medicine Seoul Korea; ^2^ Department of Biology Soonchunhyang University Asan Korea

**Keywords:** allergen, oak, pollinosis

## Abstract

**Objective:**

The component allergens from sawtooth oak, which is a main cause of tree pollinosis in Korea, have not been extensively characterized except Que ac 1. This study was undertaken to characterize the allergenic components from sawtooth oak pollen and investigate the diagnostic values of each component allergen.

**Methods:**

Transcriptomic analysis was performed to identify the birch pollen allergen homologues from sawtooth oak pollen. Recombinant Que ac 1, 2, 3, 6, 7, and 8 were produced in an *E*. *coli* expression system. IgE reactivity to each allergen was examined by ImmunoCAP and ELISA using the sera of 50 Korean tree pollinosis patients.

**Results:**

Six birch pollen allergen homologues were identified using transcriptome analysis, as follows: Que ac 1 (54.8% identity to Bet v 1), Que ac 2 (79.7% to Bet v 2), Que ac 3 (24.9% to Bet v 3), 6 (71.3% to Bet v 6), Que ac 7 (80.9% to Bet v 7), and Que ac 8 (78.9% to Bet v 8). Que ac 1 sIgE was the most frequently recognized (84.0%), followed by Que ac 2 (12.0%), Que ac 3 (6.0%), and three other allergens (2.0% each). Que ac 1 was a dominant allergen affecting 83.7% of patients suffering from allergic rhinoconjunctivitis, and 92.9% of pollen food allergy syndrome patients.

**Conclusion:**

Five novel IgE reactive components of sawtooth oak were characterized using transcriptome analysis. Que ac 1 is the single most important component allergen of sawtooth oak pollen.

## Graphical abstract

Identification of allergen homologues by transcriptome analysis of sawtooth oak pollen. Que ac 1 (54.8% identity to Bet v 1), Que ac 2 (79.7% to Bet v 2), Que ac 3 (24.9% to Bet v 3), 6 (71.3% to Bet v 6), Que ac 7 (80.9% to Bet v 7), and Que ac 8 (78.9% to Bet v 8). Production of recombinant allergens. Analysis of diagnostic values of recombinant proteins. Que ac 1 sIgE was the most frequently recognized (84.0%), followed by Que ac 2 (12.0%), Que ac 3 (6.0%), and three other allergens (2.0% each). Que ac 1 was a dominant allergen affecting 83.7% of patients suffering from allergic rhinoconjunctivitis, and 92.9% of pollen food allergy syndrome patients.

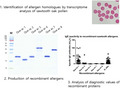

## 1 INTRODUCTION

Oak is the most important cause of seasonal pollinosis in Korea. The overall prevalence of allergic sensitization was 45.3%, and sensitization to oak was 6.6%, from 28,954 Korean adult patients in a retrospective study.^1^ Furthermore, skin reactivity to tree pollens especially to oak (4.7% to 14.4%) was significantly increased over last decade (about 0.28% annually).^2^, ^3^ However, high cross‐reactivity among tree pollens from the Fagales order (including oak, birch, alder, and hazel) often makes it difficult to identify the culprit allergen. A component‐resolved diagnosis using purified allergen molecules can help discriminate genuine sensitization from false‐positive reactions by cross‐reaction.^4^ Furthermore, more clinical information can be obtained by comparing the sensitization pattern of patients. Possible further benefits include the prediction of symptom prognosis and selection of patients who are more likely to be effectively treated by immunotherapy. Patients who are poly‐sensitized to minor allergens are less likely to be benefited by immunotherapy.

Sawtooth oak, *Quercus acutissima*, showed the strongest allergenicity among the Korean oak species.^5^ Que ac 1, which is homologous to Bet v 1, was the most potent allergen from sawtooth oak pollen. This molecule (Que ac 1) was recognized by IgE antibodies from 91.3% of the oak pollinosis patients.^6^ However, we believe that there are more allergen molecules that have yet to be identified. Only four different allergen molecules from four different oak species are officially listed in the allergen nomenclature (www.allergen.org), according to the guidelines of Allergen Nomenclature Subcommitee of the International Union of Immunological Societies, as follows: Que a 1 from *Q*. *alba* ^7^; Que m 1 from *Q*. *mongolica* ^8^; Que i 1 from *Q*. *ilex* ^9^; and Que ac 1 from *Q*. *acutissima*.^6^ Furthermore, there is only one group of protein, pathogenesis‐related protein 10 (PR‐10), from oak pollen. In contrast, there are 7 allergens of different protein families that are described from one species of birch (*Betula* *verrucosa*), as follows: Bet v 1, PR‐10^10^; Bet v 2, profilin^11^; Bet v 3, 4 EF hand polcalcin‐like protein^12^; Bet v 4, polcalcin^13^; Bet v 6, phenyl coumaran benzylic ether reductase^14^; Bet v 7, cyclophilin^15^; Bet v 8, glutathione *S*‐transferase.^16^

In this study, we used transcriptome analysis of sawtooth oak pollen to identify allergens that are homologous to birch pollen allergens. Recombinant proteins were produced, and IgE reactivities were examined using ImmunoCAP. A plausible application of the component‐resolved diagnosis of oak allergy was also evaluated.

## 2 SUBJECTS AND METHODS

### 2.1 Subjects

#### 2.1.1 Subjects and serum samples

The serum samples were collected from 50 oak pollinosis patients (average 32 years, age range 7–62 years) who visited the Allergy‐Asthma Center at Severance Hospital, Seoul, Korea (Table S1). The allergy diagnosis was based on patient history and IgE tests. Specific IgE reactivity to white oak was determined using an ImmunoCAP assay (ThermoFisher Scientific). Serum sample collection was approved by the Institutional Review Board of Severance Hospital (4–2017–1197).

## 3 MATERIALS AND METHODS

### 3.1 Transcriptome analysis of sawtooth oak pollen

Pollen grains were collected from sawtooth oak trees identified by a botanist. The total RNA was extracted with TRIzol reagent (Invitrogen) using the manufacturer's instruction. The RNA concentration, quality, and purity were assessed using a NanoDrop UV spectrophotometer (ThermoFisher Scientific) and Agilent Bioanalyzer Pico 6000 chip (Agilent Technologies) before sequencing. The ribosomal RNA was removed using the Illumina Ribo‐Zero Plus rRNA Depletion kit, and the mRNA library (Illumina) was prepared using the TruSeq stranded mRNA Library preparation kit (Illumina). The resulting library was checked with the TapeStation DNA1000 TapeScreen Assay (Agilent).

Cluster generation was performed immediately before sequencing on a cBot with the HiSeq^®^ PE Cluster Kit v4‐cBot. The sequencing was conducted using a HiSeq^®^ SBS Kit on a HiSeq^®^ 4000, operating with HiSeq Control Software v2.2.68 and base‐calling with Real Time Analysis (RTA) v1.18.66.3 (Illumina). The RNA‐seq contigs that are homologous to birch allergens (Bet v 1, 2, 3, 4, 6, 7, and 8) were analyzed.

### 3.2 Expression and purification of recombinant proteins

Que ac 2, 3, 6, 7, 8 clones were synthesized at Bioneer (Daejeon, Korea). The PCR‐amplified DNA fragments with oligonucleotide (Table 1) were subcloned into the pET6xHN‐N expression vector (Clontech Laboratories, Inc. A Takara Bio Company), which allows directional cloning, by the infusion method. The subcloned DNA sequences were determined in both directions at BioFact.

**TABLE 1:** Sequence of oligonucleotides used for directional cloning into the pET6xHN‐N expression vector

Oligonucleotide primer		Sequence^a^
Que ac 2	Forward	*TAAGGCCTCTGTCGAC*ATGTCGTGGCAAACCTACGTC
	Reverse	*CAGAATTCGCAAGCTT*TTAGAGACCCTGATCGATGAG
Que ac 3	Forward	*TAAGGCCTCTGTCGAC*ATGGCAACCAATTCAGCCCCA
	Reverse	*CAGAATTCGCAAGCTT*TCAATATTTGACAGGATATTT
Que ac 6	Forward	*TAAGGCCTCTGTCGAC*ATGGAGGCAAAAAGCAAGGTC
	Reverse	*CAGAATTCGCAAGCTT*TTAGACAAACTGATTGATAAA
Que ac 7	Forward	*TAAGGCCTCTGTCGAC*ATGTCAAATCCAAAGGTTTTC
	Reverse	*CAGAATTCGCAAGCTT*TCAACCGTGCAACTTCTCTAC
Que ac 8	Forward	*TAAGGCCTCTGTCGAC*ATGGCTGCTGCGACCGTGAAC
	Reverse	*CAGAATTCGCAAGCTT*TCAGTGCTTAGCCAAAAAGAG

^a^ Italicized sequences were included for the directional cloning by infusion technology.

Each clone was transformed into *Escherichia coli* Rosetta‐2 (DE3) (Novagen). The expression was induced by adding 1 mM isopropyl‐1‐thio‐β‐galactopyranoside. Recombinant Que ac 2 and 3 were purified from the soluble fractions. Que ac 6, 7, and 8 were purified from the inclusion bodies using Ni‐NTA resin (Qiagen), dialyzed against phosphate‐buffered saline (PBS), pH 7.4, and stored at −20°C. Recombinant Que ac 1 was also produced by the same way as described previously.^6^

In order to identify the recombinant proteins, they were confirmed by liquid chromatography‐coupled electrospray ionization‐tandem mass spectrometry (LC ESI MS/MS) after gel excision. In‐gel tryptic digestion was performed for tandem mass spectrometry at ProteomeTech.

### 3.3 IgE reactivity of recombinant allergens

Specific IgEs to the recombinant allergens were measured using the ImmunoCAP platform. For ImmunoCAP analysis, biotinylation was performed using EZ‐link^®^ Sulfo‐NHS‐LC‐Biotin (ThermoFisher Scientific). Briefly, recombinant Que ac 2, 3, 7, and 8 (2 mg) were incubated with NHS‐LC‐biotin on ice for 4 h and then dialyzed extensively against PBS to remove unreacted NHS‐LC‐biotin. Biotinylated proteins were loaded onto streptavidin ImmunoCAPs. IgE antibody binding to the proteins was measured using the Phadia UniCAP 100 system according to the manufacturer's instructions. An IgE value ≥0.35 kU/L was considered positive.

IgE reactivity to recombinant Que ac 6 was examined by ELISA, because it was not readily biotinylated due to aggregation after dialysis against PBS. The microtiter plates (Corning Inc.) were coated with recombinant Que ac 6 (2 μg/ml in 50 mM sodium carbonate, pH 9.6) and incubated overnight at 4˚C. After blocking with 3% skim milk in PBS containing 0.05% Tween 20 (PBST), serum samples (1:4 dilution in PBS) containing 1% bovine serum albumin were applied and incubated for one hour at room temperature. The IgE antibodies were detected by incubating biotinylated goat anti‐human IgE (1:1000) (Vector) for one hour, followed by incubation with streptavidin peroxidase (1:1,000) (Sigma‐Aldrich) for 30 min. Color was developed using TMB Microwell Peroxidase Substrate (SeraCare Life Science, Inc.). The absorbance at 450 nm was measured after the addition of 0.5 M H_2_SO_4_. All of the samples were duplicated. The mean absorbance plus double standard deviation of the negative controls was determined as a cutoff value.

## 4 RESULTS

### 4.1 Identification of allergen homologues by transcriptome analysis

We were able to identify 6 birch allergen homologous molecules (Table 2). Que ac 1 (GenBank accession No. MN201198), 2 (MW506866), 3 (MW50686), 6 (MW506868), 7 (MW506869), and 8 (MW506870) share 54.8% identity to Bet v 1, 79.7% to Bet v 2, 24.9% to Bet v 3, 71.3% to Bet v 6, 80.9% to Bet v 7, and 78.9% to Bet v 8, respectively. However, a Bet v 4 homologous molecule was not found.

**TABLE 2:** Allergen homologues obtained from sawtooth oak pollen transcriptomes

Biochemical identity	Allergen	Species	Sequence identity	Seq number	QueryID^a^
Pathogenesis‐related protein 10 (Que ac 1)^b^ <17 kDa>	Bet v 1 Que a 1 Que m 1	*Betula verrucosa* *Quercus alba* *Quercus mongolica*	54.8% 73.1% 71.9%	8	001836, 002011, 007377, 008550, 008759, 014073, **007378**, 013296,
Profilin (Que ac 2), <15 kDa>	Bet v 2 Amb a 8 Mal d 4 Ara h 5	*Betula verrucosa* *Ambrosia artemisiifolia* *Malus domestica* *Arachis hypogaea*	79.7% 72.9% 80.2% 87.8%	3	**003306** , 008147, 015060,
Polcalcin‐like protein (Que ac 3) <24 kDa>	Bet v 3 Ole e 8	*Betula verrucosa* *Olea europaea*	24.9% 53.8%	1	**009974** ,
Phenyl coumarin benzylic ether reductase (Que ac 6) <35 kDa>	Bet v 6	*Betula verrucosa*	71.3%	6	011586, **016135**, 007675, 008742, 008361, 012356,
Cyclophilin (Que ac 7) <18 kDa>	Bet v 7	*Betula verrucosa*	80.9%	1	**003618** ,
Thaumatin‐like protein <30 kDa>	Jun a 3 Pru av 2 Mal d 2	*Juniper ashei* *Prunus avium* *Malus domesticus*	47.6% 49.4% 48.6%	3	**012441** , 014967, 008403,
Glutathione *S*‐transferase <23 kDa>	Bet v 8	*Betula verrucosa*	78.9%	8	002338, 002726, 009783, 011000, 013494, 014046, **014047** , 016914,

^a^ Sequences with full‐sequence information are underlined.

^b^ Putative names of sawtooth oak allergens are in the brackets.

### 4.2 IgE reactivity and diagnostic sensitivity of the recombinant allergens

**FIGURE 1:**
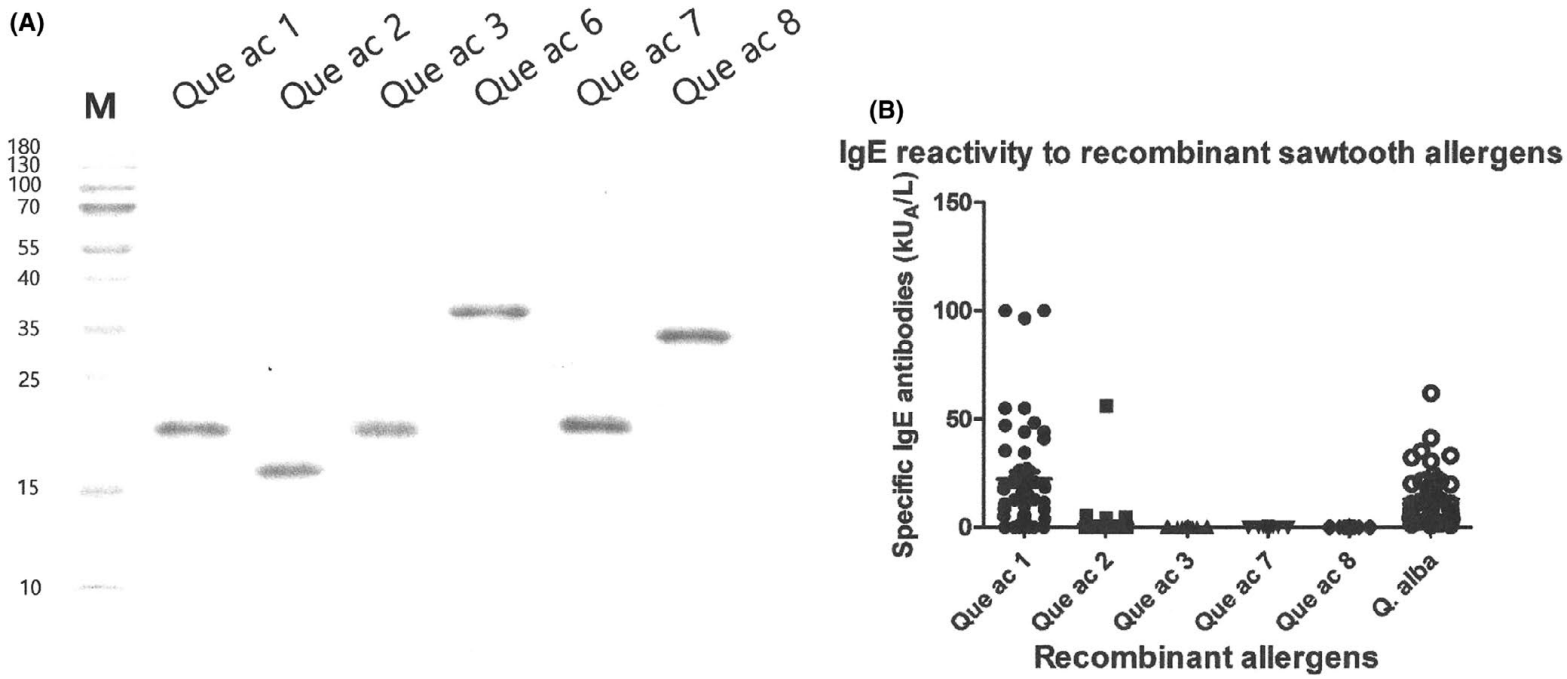
IgE reactivity to component allergens from sawtooth oak. Produced recombinant allergens was run on 15% SDS‐PAGE (A), and IgE reactivity to each allergen was assessed using the ImmunoCAP system (B)

Six recombinant allergens were produced using the same expression system; pET6xHN‐N vector and *E*. *coli* Rosetta‐2 (DE3) (Figure 1). The molecular identity of the recombinant proteins was confirmed again using LC ESI MS/MS (Table 3). Que ac 1 was the most frequently recognized by serum IgE (84.0%), followed by Que ac 2 (12.0%), Que ac 3 (6.0%), and the other three allergens (2.0% each). Only the Que ac 2 allergen was recognized by serum IgE from two patients who were not sensitive to Que ac 1 and increased the diagnostic sensitivity by 4.0% (Table 4). None of the other allergens added diagnostic value to Que ac 1. One of Que ac 2 sensitive patients suffered from allergic rhinitis and PFAS, while one only had allergic rhinitis.

**TABLE 3:** Identification of recombinant proteins by tandem mass analysis

Recombinant protein	NCBI Blast	Protein name	Species	Mass (Da)	Mascot score
Que ac 1	AFF59686.1	Bet v 1 allergen‐like	*Quercus rubra*	17,359	223
	PON61986.1	Bet v 1‐type allergen	*Parasponia andersonii*	17,566	168
	XP_018825721.1	Major allergen Pru ar 1‐like	*Juglans regia*	17,592	167
	ACJ23862.1	Cas s 1 pollen allergen	*Castanea sativa*	17,503	166
Que ac 2	AAW69549.1	Profilin, Cuc m 2	*Cucumis melo*	14,029	3,472
	Q9LEI8.1	Pollen allergen Hev b 8.0204	*Hevea brasiliensis*	14,112	3,463
	Q64LH1.1	Pollen allergen Amb a 8	*Ambrosia artemisiifolia*	14,218	2,556
Que ac 3	XP_023880949.1	Probable calcium‐binding protein CML27	*Quercus suber*	19,753	294
	PHU24259.1	Calcium‐binding allergen Ole e 8	*Capsicum chinense*	18,462	83
	XP_008356663.1	Probable calcium‐binding protein CML27	*Malus domestica*	19,137	51
Que ac 6	XP_023927210.1	Isoflavone reductase homolog PCBER‐like	*Quercus suber*	34,402	386
	XP_010536348.1	Isoflavone reductase homolog P3‐like	*Tarenaya hassleriana*	34,430	122
	XP_021644235.1	Isoflavone reductase‐like protein	*Hevea brasiliensis*	33,240	106
Que ac 7	POE69746.1	Peptidyl‐prolyl cis‐trans isomerase cyp 19–3	*Quercus suber*	49,763	638
	XP_018823166.1	Peptidyl‐prolyl cis‐trans isomerase cyp 19–3	*Juglans regia*	19,116	267
	AGI78541.1	cyclophilin	*Suaeda sala*	19,091	185
Que ac 8	XP_023893302.1	Gutathione *S*‐transferase L3‐like	*Quercus suber*	27,086	468
	XP_008359696.2	Gutathione *S*‐transferase L3‐like	*Malus domestica*	28,439	156

**TABLE 4:** Clinical patient characteristics and IgE reactivity to component allergens

Characteristics	No. of patients (%)	IgE reactivity to component allergens (%)						
		Que ac 1	Que ac 2	Que ac 3	Que ac 6	Que ac 7	Que ac 8	None
Sex/age (average 32.6)								
Male (average 29.4 ± 13.3 years)	29 (58.0)	24 (82.8)	5 (17.2)	2 (6.9)	1 (3.5)	0 (0.0)	0 (0.0)	3 (10.3)
Female (average 37.0 ± 16.7 years)	21 (42.0)	18 (85.7)	1 (4.8)	1 (4.8)	0 (0.0%)	1 (4.8)	1 (4.8)	3 (14.3)
Comorbidities								
Allergic rhinitis	48 (96.0)	40 (83.3)	6 (12.5)	3 (6.3)	1 (2.1)	1 (2.1)	1 (2.1)	6 (12.5)
Allergic conjunctivitis	8 (16.0)	7 (87.5)	0 (0.0)	0 (0.0)	0 (0.0)	0 (0.0)	0 (0.0)	1 (12.5)
Rhinoconjunctivitis	49 (98.0)	48 (98.0)	6 (12.2)	3 (6.1)	1 (2.0)	1 (2.0)	1 (2.0)	1 (2.0)
Pollen food allergy syndrome	14 (28.0)	13 (92.9)	1 (7.1)	0 (0.0)	0 (0.0)	0 (0.0)	0 (0.0)	0 (0.0)
Asthma	11 (22.0)	10 (91.0)	1 (9.1)	1 (9.1)	0 (0.0)	0 (0.0)	0 (0.0)	1 (9.1)
Atopic dermatitis	2 (4.0)	2 (100.0)	1 (50.0)	1 (50.0)	1 (50.0)	1 (50.0)	1 (50.0)	0 (0.0)

Notably, IgE reactivity to Que ac 1 was even stronger than IgE reactivity to *Q*. *alba* (t7) pollen extract, which is currently used for the diagnosis of oak allergy (Figure 1B).

A possible change in IgE reactivity by the structural change of Que ac 3 induction was examined by ELISA with 10 mM CaCl_2_. However, there was no significant change in IgE reactivity (data not shown).

### 4.3 Sensitization profile and disease entities

The most common symptom from oak allergy was allergic rhinosinusitis (83.7%) (Table 4). Among allergic rhinosinusitis patients, 40 (83.3%) were sensitized to Que ac 1, 7 (15.6%) to Que ac 2, 3 (6.3%) to Que ac 3, 1 to (2.1%) to Que ac 6, 1 to (2.1%) to Que ac 7, and 1 to (2.1%) to Que ac 8. Six patients’ sera showed no reactivity to any of the six allergens.

Fourteen (28.0%) patients had pollen food allergy syndrome (PFAS) and 13 (92.9%) were sensitized to Que ac 1. Only one patient with PFAS was solely sensitized to Que ac 2 without sensitization to Que ac 1. Two PFAS patients were sensitized to both Que ac 1 and 2.

## 5 DISCUSSION

We identified five new recombinant component allergens from sawtooth oak pollen (which is the primary sensitizer in Korea) in order to investigate the application of CRD in the diagnosis of oak allergy.

Not surprisingly, Que ac 1 was recognized by serum IgE in 84.0% of patients. Only two more patients (4.0%) could be diagnosed by Que ac 2, and no diagnostic value was credited to the other allergens. Que ac 1 was the single most potent allergen, although it had no statistical correlation with a specific disease entity. Most patients (76.0%) were sensitized to one component allergen, 5 (10.0%) to two, 1 (2.0%) to 5, and 6 (12.0%) to none. Three more positives (90.0%) could be diagnosed if we adapted 0.1 kU_A_/L as cutoff value.^17^ It is plausible that the inclusion of some Que ac 1 isoforms could increase the diagnostic sensitivity since various Que ac 1 isoforms were described.^6^ Six patients (12%) demonstrated no sensitization to the component allergens test. The possibility of false positivity to white oak, which is not native to Korea, cannot be ruled out due to some cross‐reactivity between carbohydrate determinant (CCD) and panallergens. CCD often causes false‐positive IgE reactions without a clinical manifestation.^18^

Sensitization to a minor allergen component can also cause cross‐reactivity. In particular, sensitization to polcalcin may lead to a diagnosis of allergy mirage.^19^ One of the three Que ac 3 sensitized patients had positive skin tests to various trees, grasses, and weed pollens.

Species‐specific or component allergen Que ac 1‐specific sensitization indicates primary sensitization and is most likely to be benefited by immunotherapy. However, sensitization to panallergens such as Que ac 2 (profilin), Que ac 3 (polcalcin‐like), or Que ac 8 (glutathione *S*‐transferase) without sensitization to Que ac 1 implies non‐genuine sensitization and is less likely to be suitable for immunotherapy. Notably, up to half of profilin sensitized patients suffer from PFAS to tomato, melon, watermelon, and citrus.^17^, ^20^ Sensitization to profilin is also known to be associated with grass pollinosis.^21^ Among the five minor allergen homologues from sawtooth oak pollen, only Que ac 2 meets the inclusion criteria for a novel allergen. It is officially listed in the WHO/international union of the immunological societies allergen nomenclature subcommittee according to the guideline.^22^ Poor recognition of oak minor allergens, compared to birch allergens, may reflect the primary sensitization to oak pollens in Korea. Relatively higher percentages of sensitization to birch minor allergens were reported; 22% for Bet v 2, 10% for Bet v 3, 5% to Bet v 4, 32% to Bet v 6, 21% to Bet v 7, and 13% to Bet v 8. Studies on the cross‐reactivity between these minor allergens from oak and birch allergens would be interesting. It would be useful if oak‐specific or birch‐specific allergen is characterized. Different fruit consumption pattern and different regional flora should be taken into account for better diagnosis of PFAS. Sensitization to PR‐10 molecules is mainly responsible for PFAS in Korea.^23^ Furthermore, peanut and apple are described as the most common culprit foods causing anaphylaxis in PFAS in Korea, indicating the possible role of Que ac 1, a primary sensitizer, which is homologous to Bet v 1.^24^ Molecular characterization of PR‐10 molecules from commonly consumed Korean fruits, such as Korean melon, ginseng, sesame leaf, deodeok, and lotus root,^25^ should be done to understand PFAS in more detail. PFAS in Korea should be dependent on the cross‐reaction of Que ac 1 with homologous allergens from commonly consumed fruits. Bet v 1 and Que ac 1 may have different cross‐reactivity to fruit allergens.

In conclusion, we characterized five new IgE reactive components (Que ac 2, 3, 6, 7, and 8) from sawtooth oak pollen and investigated their diagnostic value. Que ac 1 and Que ac 2 are useful to make a diagnosis of oak pollinosis. Most IgE reactivities are directed to Que ac 1. However, no significant sensitization pattern was correlated to any allergic diseases. The recombinant allergens that were identified in this study will be useful to improve allergy diagnostics.

## Supporting information

Table S1

## Data Availability

The data that supports the findings of this study are available in the supplementary material of this article.
