# Supplementary material for: Sensitization profile to sawtooth oak component allergens and their clinical implications
Source: J Clin Lab Anal. 2021 May 18;35(7):e23825. doi: 10.1002/jcla.23825 (PMC8274986; doi:10.1002/jcla.23825)
Supplement: Supplementary file 1 — Table S1 [file JCLA-35-e23825-s001.docx]

| Supplementary Table. Clinical features of the enrolled patients and IgE reactivity to component allergens | | | | | | | | | | | | |
| --- | --- | --- | --- | --- | --- | --- | --- | --- | --- | --- | --- | --- |
| **No.** | Gender/age | Diagnosis* | Co-sensitization profile** | IgE to allergens (kU_A_/l) | | | | | | | | Absorbance at 450 nm |
|  |  |  |  | IgE to birch (t3) | IgE to oak (t7) | total IgE | Que ac 1 | Que ac 2 | Que ac 3 | Que ac 7 | Que ac 8 | Que ac 6 |
| 1 | M/38 | AR | t2, d2, g5, g6 | 18 | 3.59 | 166 | **11.5** | 0 | 0 | 0 | 0 | 0.1253 |
| 2 | M/16 | AR | w1, m6, d2, e5 | 71 | 30.7 | ND*** | **55** | 0.14 | **0.43** | 0.13 | 0.07 | 0.13275 |
| 3 | M/13 | AR | w1, e5 | 39.5 | 19.7 | 492 | **26.4** | 0.01 | 0.01 | 0.03 | 0.04 | 0.1332 |
| 4 | M/16 | AR | g6, d2, e1 | 4.01 | 3.8 | 4.01 | **2.16** | 0 | 0 | 0 | 0 | 0.1266 |
| 5 | M/9 | AR | w1, f13 | 3.34 | 1.27 | 52.4 | 0 | **5.44** | 0.13 | 0.06 | 0.04 | 0.1299 |
| 6 | F/45 | AR, AS | d2, e1, e5 | 25 | 10.9 | ND | **8.26** | 0.03 | 0.01 | 0.02 | 0.02 | 0.1198 |
| 7 | F/7 | AR | w1, d2 | 2.63 | 5.22 | 2059 | 0.08 | 0.08 | 0.06 | 0.07 | 0.06 | 0.1183 |
| 8 | M/11 | AR | g6, w1, w22, d2, f13 | 22.8 | 33.2 | ND | **12.9** | 0.08 | 0.08 | 0.1 | 0.1 | **0.168** |
| 9 | M/16 | AR | w1 | 14.7 | 16.7 | ND | **16.5** | 0.25 | 0.01 | 0.06 | 0.01 | 0.12095 |
| 10 | F/61 | AR, BA | d2 | ND | 4.56 | 1404 | 0.016 | 0.018 | 0.012 | 0.014 | 0.015 | 0.1215 |
| 11 | M/38 | AR, PFAS | d1, d2, g5, t1, t2, g8, g5, g6, d1, d2, e1 | 9.4 | 1.71 | 35.1 | **3.76** | 0 | 0 | 0 | 0 | 0.127 |
| 12 | M/30 | AR, AS, PFAS | w1, d2 t1, t2, t8, t10, t11, t12, t15, g8, g6, w22 | 79.2 | 62 | 871 | **>100** | 0.02 | 0.01 | 0.04 | 0.17 | 0.12215 |
| 13 | M/20 | AR, AS | f4, t1, t2, g2, g3, g5, w1, w5, w8, w10, w9, w22, m1, e1, e5, f4 | ND | 5.31 | ND | **9.92** | 0 | 0 | 0 | 0.03 | 0.1192 |
| 14 | F/47 | AR, BA, PFAS | t1, t2, t8, t10, t11, t12, t70, g8, g2, w8, m3, m5, d72, e4, e6, i6, i206 | 16.7 | 12.7 | 306 | **26.2** | 0.01 | 0 | 0.09 | 0.04 | 0.1229 |
| 15 | F/54 | AR | NA | 19.1 | 3.24 | 42.6 | **5.55** | 0 | 0 | 0 | 0 | 0.1158 |
| 16 | F/26 | AR, DA, PFAS | f95, f84, f44, f242 | ND | 13.1 | ND | **19.7** | 0 | 0 | 0.02 | 0.03 | 0.11805 |
| 17 | F/34 | AR | d2, e5, t215 | 20 | 11.7 | ND | **15.7** | 0.03 | 0.02 | 0.05 | 0.03 | 0.12625 |
| 18 | M/45 | AR, BA | w6, d2 t8, t10, t11, t205, t15, t19, g8, g2, g3, g5, g6, w5, w10, w9, w22, w19, d1, d2, e1, e5, e3, e71, f11, f4 | ND | 11.2 | MD | **20.3** | **0.67** | 0.06 | 0.09 | 0.11 | 0.13205 |
| 19 | M/31 | AR, BA | t2, d2 t1, t2, t8, t10, t11, t12, t70, t205, t15, t213, g8, g2, g3, g5, g6, w1, w5, w8, w10, w22, m5, d72, d2, e1, e5, e3, e84, i6, i206, | 36.8 | 21.8 | 179 | **22.7** | 0.06 | **0.64** | 0.03 | 0.03 | 0.12175 |
| 20 | F/54 | AR | ND | 32.8 | 6.36 | ND | **34.6** | 0 | 0 | 0.01 | 0.01 | 0.11665 |
| 21 | M/34 | AR, ARC | t1, g5, d2 | ND | 3.29 | ND | 0 | 0.19 | 0.02 | 0.07 | 0.01 | 0.11525 |
| 22 | F/21 | AR, ARC | d2 | 5.41 | 1.03 | 351 | **2.43** | 0.02 | 0 | 0.02 | 0.02 | 0.11445 |
| 23 | M/13 | AR, PFAS | d2 t1, t2, t8, t10, t11, t12, t70, t205, t15, t19, t213, g8, g2, g3, g5, g6, w1, w10, w9, w19, d2, e1, e5, f11 | 10.5 | 20.2 | 32.7 | **44** | **0.68** | 0.01 | 0.02 | 0.02 | 0.11445 |
| 24 | M/21 | AR, PFAS | d2,g5, e5, t1, t2, t8, t10, t11, t12, t70, t205, t15, t19, t213, g8, g2, g3, g5, g6, w5, w8, w9, w22, d1, d2, e1, e71, e82, i206, k82, f11, | 44.6 | 21.4 | ND | **55.3** | 0.12 | 0.07 | 0.09 | 0.08 | 0.119 |
| 25 | M/35 | AR, PFAS | d1, d2, t2, t8, t17, w12 | ND | 8.97 | ND | **18.6** | 0 | 0 | 0 | 0.04 | 0.122 |
| 26 | M/62 | AR | w1,f49, g6,w6 | ND | 5.04 | ND | **13.5** | 0.17 | 0 | 0 | 0 | 0.1222 |
| 27 | M/36 | AR | d2, d1, e71, e73, e74. w1,w6,w22, | 0.34 | 4.11 | ND | 0.16 | 0.09 | 0.07 | 0.08 | 0.07 | 0.123 |
| 28 | M/42 | AR, PFAS | d1, d2, e1, t1, t2, t70, t213, g5, w1, w8, d72, e1, e5 | 7.23 | 4.84 | 248 | **8.54** | 0.03 | 0.07 | 0.01 | 0.02 | 0.113 |
| 29 | F/56 | AR | w1,w6,d2,i6 | 29.8 | 5.92 | ND | **15.9** | 0 | 0 | 0.01 | 0 | 0.1167 |
| 30 | F/61 | CU | w1,w6,d2,f4 | ND | 3.88 | ND | **2.61** | 0.03 | 0.03 | 0.04 | 0.03 | 0.12165 |
| 31 | F/24 | AS | d1, d2, m70, w22, d1, d2, t1, t8, t10, t15, w12, m5 | 8.54 | 8.52 | 383 | **17** | 0.05 | 0.03 | 0.05 | 0.05 | 0.1126 |
| 32 | M/29 | AR, PFAS | f95,f49 | 42 | 19.9 | 30.6 | **11** | 0 | 0 | 0.01 | 0 |  |
| 33 | M/32 | AR, PFAS | w1,w6,d2 t2, t8, t10, t12, t70, t213, t17, g8, g2, g3, w1, w5, w8, d1, d2, e1, f11 | 36.6 | 6.26 | 377 | **21** | 0.03 | 0.02 | 0.02 | 0.03 |  |
| 34 | F/53 | AR | t1, t2, t70, g8, w22, w19, m1, m2, m3,m5 d1, d2, e1, e5, e4, e73, f9 | 38.7 | 35.2 | ND | **47.1** | 0.01 | 0.04 | 0.01 | 0.04 | 0.1039 |
| 35 | F/16 | AR, ARC, PFAS | t2, t15 | 20.6 | 18.5 | ND | **17.8** | 0 | 0 | 0.02 | 0.02 | 0.11785 |
| 36 | M/58 | AR, AS, PFAS |  | 5.94 | 3.38 | ND | **5.21** | 0 | 0 | 0.03 | 0.02 | 0.1272 |
| 37 | F/20 | BA, AR, ARC, PFAS, CSU, AD | d1, d2, e1, e5 | 3.29 | 5.27 | 467 | **35.4** | 0.1 | 0.03 | 0.06 | 0.06 | 0.10815 |
| 38 | F/42 | AR | d1, d2, w6, w22 | 13.2 | 21.3 | ND | **44** | 0.03 | 0.01 | 0.04 | 0.04 |  |
| 39 | F/23 | ARC, FA | d1, d2, w22 | 59.9 | 41.3 | 136 | **>100** | 0 | 0 | 0.01 | 0.02 | 0.1209 |
| 40 | M/35 | AR | d1,d2,m6,e1,e5 | ND | 7.94 | ND | **19.3** | 0.13 | 0.09 | 0.1 | 0.15 | 0.1246 |
| 41 | F/32 | AR, BA | d1, e5, t2 | 5.73 | 2.5 | 839 | **5.65** | 0.06 | 0.04 | 0.06 | 0.07 | 0.10185 |
| 42 | M/27 | AR, ARC | d1, d2 | 31.2 | 24.3 | 603 | **96.5** | 0.02 | 0.01 | 0.05 | 0.05 | 0.1058 |
| 43 | M/15 | AR, PFAS | w6, f49, f95 | 17.5 | 10.5 | ND | **55.1** | 0.03 | 0.01 | 0.02 | 0.02 | 0.1066 |
| 44 | M/38 | AR | w1, w6 | 13.3 | 6.52 | 56.8 | **14.5** | **4.36** | 0.01 | 0.02 | 0 | 0.11425 |
| 45 | M/38 | AR, CSU | w1,w6,w22,d2 | 1.19 | 12.6 | 1223 | 0.06 | 0.05 | 0.04 | 0.07 | 0.07 | 0.1205 |
| 46 | F/19 | AR | t11,t12,w8,f25 | 0.52 | 0.92 | 1589 | 0.3 | 0.05 | 0.04 | 0.05 | 0.06 | 0.1187 |
| 47 | F/29 | AR, AD, PFAS | d2 | 17.8 | 8.13 | >5000 | **12.8** | **4.95** | **0.4** | **0.59** | **0.64** | 0.10325 |
| 48 | F/52 | AR, AS, DA | w6,i6 | ND | 32.3 | ND | **48.3** | 0.02 | 0.02 | 0.09 | 0.05 | 0.0971 |
| 49 | M/22 | AR, ARC, DA | w22,d2,f95 | 20.10 | 21.70 | 223 | **40.9** | 0.01 | 0 | 0.01 | 0.02 | 0.0943 |
| 50 | M/32 | AR | d2, f24 | 35.00 | 17.50 | 1295 | **27** | **0.04** | 0.03 | 0.05 | 0.07 | 0.1083 |

*Abbreviation: AD, Atopic dermatitis; AR, Allergic rhinitis; ARC, Allergic rhinoconjuctivitis; AS, Asthma; BA, Bronchial asthma; CC, Chronic cough; CR, Chronic rhinitis; CU, Chronic urticarial; CV, Conjunctivitis; DA, Drug allergy; FA, Food allergy; PFAS, Pollen food allergy syndrome.
**d1, *Dermatophagoides pteronyssinus*; d2, *D. farinae*; d72, *Tyrophagus putrescentiae*; e1, Cat dander; e2, Dog hair; e5, Dog dander; e71, Mouse epithelium; e83, Swine epithelium; f4, Wheat; f5, Rye; f6, Barley; f9, Rice; f11, Buckwheat; f12, Pea; f13, Peanut; f14, Soybean; f25, Tomato; f33, Orange; f47, Garlic; f 48, Onion; f95, Peach; f208, Lemon; g1, Sweet vernal grass; g2, Bermuda grass; g3, cocksfoot; g5, Rye-grass; g6, Timothy grass; g7, Common reed; g8, Meadow grass; g12 Cultivated Rye; g15, Cultivated wheat; g201, Barley; i6, German cockroach; i206, American cockroach; k84, Sunflower seed; m1, *Penicillium chrysogenum*; m2, *Cladosporium herbarum*; m3, *Aspergillus fumigatus*; m5, *Candida albicans*; m6, Alternaria; t1, Acer; t2, Alder; t3, Birch; t4, Hazel; t5, Beech; t7, Oak; t8, Elm; t10, Walnut; t11, Platanus; t12, Willow; t15, White ash; t17, Japanese cedar; t70, Mulberry; t205, Elder; w1, Common ragweed; w4, False ragweed; w5, Wormwood; w6, Mugwort; w7, Chrysanthemum; w8, Dandelion; w9, Plantago; w10, Goosefoot; w11, Russian thistle; w12, Goldenrod; w16, Rough marsh elder; w22, Japanese hop.

***ND, Not determined.
